# Suppression of ROS Production by Exendin-4 in PSC Attenuates the High Glucose-Induced Islet Fibrosis

**DOI:** 10.1371/journal.pone.0163187

**Published:** 2016-12-15

**Authors:** Ji-Won Kim, Shin-Young Park, Young-Hye You, Dong-Sik Ham, Seung-Hwan Lee, Hae Kyung Yang, In-Kyung Jeong, Seung-Hyun Ko, Kun-Ho Yoon

**Affiliations:** 1 Division of Endocrinology and Metabolism, Department of Internal Medicine, College of Medicine, The Catholic University of Korea, Seoul, Korea; 2 Convergent Research Consortium for Immunologic Disease, Catholic Research Institutes of Medical Science, The Catholic University of Korea, Seoul, Korea; 3 Division of Endocrinology & Metabolism, Department of Internal Medicine, Kyung Hee University School of Medicine, Seoul, Korea; CHA University, REPUBLIC OF KOREA

## Abstract

Pancreatic stellate cells (PSCs) play a major role to fibrotic islet destruction observed in diabetic patients and animal model of diabetes. Exendin-4 (Ex-4) is a potent insulinotropic agent and has been approved for the treatment of type 2 diabetes. However, there have been no reports demonstrating the effects of Ex-4 on pancreatic islet fibrosis. In this study, Ex-4 treatment clearly attenuated fibrotic islet destruction and improved glucose tolerance and islet survival. GLP-1 receptor expression was upregulated during activation and proliferation of PSCs by hyperglycemia. The activation of PKA pathway by Ex-4 plays a role in ROS production and angiotensin II (Ang II) production. Exposure to high glucose stimulated ERK activation and Ang II-TGF- β1 production in PSCs. Interestingly, Ex-4 significantly reduced Ang II and TGF-β1 production by inhibition of ROS production but not ERK phosphorylation. Ex-4 may be useful not only as an anti-diabetic agent but also as an anti-fibrotic agent in type 2 diabetes due to its ability to inhibit PSC activation and proliferation and improve islet fibrosis in OLETF rats.

## Introduction

Pancreatic β-cell failure plays an important role in the progression and development of type 2 diabetes (T2DM) [1]. In addition to progressive β-cell dysfunction, the pancreatic islet architecture of patients with type 2 diabetes shows variable morphologic changes, such as islet fibrosis and β-cell loss [2]. Islet fibrosis could be an important outcome of progressive β-cell failure because it may accelerate β-cell destruction, such as that in chronic pancreatitis, or disrupt β-cells [3]. It has also been proposed that islet fibrosis is present in the late stage of β-cell dysfunction during the progression to T2DM [4].

Pancreatic stellate cells (PSCs) are involved in the progression of pancreatic islet fibrosis in T2DM [5]. PSCs are important in the pancreatic fibrogenesis that is associated with chronic pancreatitis [6]. PSCs have been identified as the extracellular matrix (ECM) proteins found in pancreatic fibrosis or chronic pancreatitis both experimental animals as well as humans [7, 8]. During the quiescent stage, PSCs contain numerous lipid droplets in the cytoplasm [9]. When activated by inflammatory cytokines or oxidative stress, PSCs transform into myofibroblast cells, which can be recognized by immunostaining for α-smooth muscle actin (α-SMA) [7, 10]. PSCs show distinctly increased ECM protein synthesis in response to growth factors and cytokines and and platelet-derived growth factor and transforming growth factor-β exert potent proliferative effects on PSCs [11]. We found that pancreatic stellate cells are activated and proliferate when exposed to high concentrations of glucose and insulin via the Ang II type 2 receptor signaling pathway, though the exact mechanisms underlying this phenomenon remain to be confirmed. Treatment with an ACE inhibitor suppressed ECM protein expression in PSCs, and these effects were accompanied by the down-regulation of α-SMA [8]. This finding suggests that islet fibrosis and PSC proliferation are related to the renin–angiotensin system (RAS). Taken together, PSCs play a major role in fibrotic islet destruction in patients with T2DM and that suppressing the activation and proliferation of PSCs is a potential mechanism through which the progression of T2DM may be delayed or prevented.

Glucagon-like peptide-1 (GLP-1) is an incretin hormone that is known to have an insulinotropic action. Interestingly, Exendin-4 (Ex-4), a GLP-1 analogue, has been shown to improve β-cell function by up-regulating the expression of key genes involved in insulin secretion [12]. Ex-4 treatment also prevented the development of diabetes in a partial pancreatectomy rat model of T2DM, and resulted in a 40% expansion of β-cell mass due both to the differentiation and neogenesis of precursor cells and to enhanced β-cell proliferation [13, 14]. Furthermore, Ex-4 has anti-inflammatory and anti-hypertensive effects and acts as an anti-fibrotic agent in mesangial cells [15, 16]. Ex-4 inhibited the proliferation of human mesangial cells and down-regulated the high-glucose induced expression of TGF-β1 and connective tissue growth factor [17]. GLP-1 and Ex-4 activate multiple signaling pathways such as cAMP/PKA, phosphatidylinositol 3-kinase and mitogen-activated protein kinase (MAPK), which lead to the islet mass increase and β-cell growth [18, 19]. However, because the role of Ex-4 in PSCs remains to be elucidated, we investigated the effects of Ex-4 on activated PSCs under high-glucose conditions.

## Materials and Methods

### Animal experiments

Thirty-week-old male otsuka long-evans tokushima fatty (OLETF) rats were housed in a specific pathogen-free facility and maintained under a 12 h light/12 h dark cycle, with free access to standard rodent chow and water, except when fasting. Thirty-week-old OLETF rats were randomly allocated to control, insulin and Ex-4 treatment groups by IPGTT: the saline group (n = 10, control group); insulin group (n = 10, glucose-matched control group of Ex-4 group); or Ex-4 group (2.5 nmol/kg/every two days, n = 10). The insulin treatment group was intraperitoneally injected with insulin daily for 12 weeks, the Ex-4 group was intraperitoneally administered long-acting Ex-4 (LAPS-exendin-4, HM11260C, Hanmi Research Center) twice per day for 12 weeks, and the control group was given saline on the same schedule as the insulin-administered group. The animals’ overnight fasting serum glucose levels were measured daily using a glucometer (ARKRAY Inc.); the animals’ body weights were also measured daily. After 12 weeks of treatment, the rats were sacrificed for physiological and histological analyses. The Animal Care Committee of The Catholic University (IACUC: approved No. 2013-0028-02) of Korea approved the experimental protocol, and all experiments were performed in accordance with relevant guidelines and regulations.

### Intraperitoneal glucose tolerance test (IPGTT)

The intraperitoneal glucose tolerance test (IPGTT) was performed in rats aged 33, 36, 39, and 42 weeks. Briefly, 2 g/kg body weight glucose was injected intraperitoneally after overnight fasting, and the blood glucose level was measured with a glucometer before injection and at 30, 60, 90, and 120 min after injection. The area under the glucose curve (AUCg) was calculated.

### Masson’s trichrome staining

We calculated the percentage of fibrosis areas (blue) within islets by planimetry. We used an image analyzer (Optimas 6.51, Media Cybertics, Tempe, AR). The amount of fibrosis was presented as a percentage of the total islet area and was calculated from a mean of islets per pancreas: (area of fibrosis/total area of islets) X 100.

### β-cell mass in pancreatic islet

The relative β-cell volumes were quantified by the point counting method under the microscope (Olympus AX70, Tokyo, Japan) connected to a camera equipped with a color monitor with 100-point transparent overlay [20]. Briefly, pancreas specimen stained with anti-insulin primary antibody were observed under the microscopy of 200 X magnifications and positioned under a regular lattice overlaid on a monitor. The relative β-cell volume in the pancreatic tissue was described as the number of points corresponding to the anti-insulin antibody-stained area/number of points corresponding to remaining pancreatic area. β-cell mass was calculated by multiplying the relative percentage of β-cells by the weight of total pancreatic tissue [21]. An average of 207.8 fields and 20784.4 points in non-overlapping fields were counted systematically from each section with 5 sections being selected per tissue block.

### PSC isolation and culture

Rat PSCs were isolated as detailed previously using the method described by Apt et al. with some modifications [6]. Rat PSCs were isolated from Sprague-Dawley rsts (200–250 g) by digesting the pancreatic duct with collagenase P in HBSS. The viability of the isolated cells was assessed using the trypan blue exclusion method, and the cells were counted using a hemocytometer. The isolated PSCs were cultured in DMEM (Gibco) supplemented with 17.5 mM D-glucose, 10% FBS, and antibiotics (1,000 IU/mL penicillin, 1,000 mIU/mL streptomycin, Gibco) at 37°C in a humidified 95% air/5% CO_2_ atmosphere. At confluence, the cells were harvested and re-seeded at equal densities for use in the experiments described below. All experiments were performed using culture-activated cells (passages 2–5).

### Treatment of PSCs with high glucose and Ex-4

Cultured rat PSCs were seeded at equal densities in DMEM containing 10% FBS and 5.5 mM glucose. After serum starvation for 24 h, the cells were treated with a low (5.5 mM D-glucose) or high (27.7 mM D-glucose) level of glucose with or without 10 nM Ex-4 (E7144, Sigma-aldrich) for 3 or 9 days.

### Immunostaining

PSCs were fixed with 4% paraformaldehyde (PFA) at room temperature for 10 min. After washing with phosphate-buffered saline (PBS), the fixed cells were stored at 4°C until staining. To block non-specific binding sites, 10% normal donkey serum in PBS was applied for 30 min. The cells were then incubated overnight with a primary antibody against insulin (1:200, clone Z006, Zymed), α-SMA (1:400, A2547, Sigma-Aldrich) or GLP-1R (1:50, ab39072, Abcam). After washing, the cells were incubated with FITC-conjugated donkey anti-mouse antibody (1:200, Jackson ImmunoResearch Laboratories) in PBS for 1 h at room temperature in the dark. The cells were then washed with PBS and mounted in an anti-fade medium containing DAPI. The cells were observed under an inverted fluorescence microscope. To confirm the oxidative stress-induced DNA damage in the islets, we used a monoclonal antibody to 8-hydroxy-29-deoxyguanosine (8-OHdG, 1:100, Abcam, Cambridge, MA). The sections were treated with microwave irradiation for 15 min in 10 mM citric buffer (pH 6.0) for antigen retrieval. After incubation with horse serum for 30 min to block non-specific reactions, the primary antibody was applied overnight at 4°C. They were then incubated with biotinylated horse anti-mouse IgG antibody with peroxidase-conjugated streptavidin labeling reagent (1:100; Vector Laboratories, Burlington, ON, Canada) as the secondary antibody. We randomly selected two slides per groups. Specimen was double stained with using trichrome reagent and 8-OHdG antibody. We counted the nuclei in the fibrotic area of islets (around 150 um) then we also counted the cells positive for 8-OHdG. 14 islets from saline-treated group, 12 islets from insulin-treated group, and 10 islets from exendin-4-treated group (Ex-4) were utilized in the analysis.

### RT-PCR

Total RNA from PSCs cultured under the conditions described above was extracted with Trizol Reagent (Invitrogen). RNA (1 μg) was reverse transcribed into cDNA using oligo(dT)12–18 primers with SuperScript®III (Invitrogen) at 42°C for 1 h, followed by incubation at 72°C for 15 min, in standard buffer. The relative gene expression levels were determined with reference to the expression of the GAPDH gene. Gene-specific primers were designed based on published sequences. The PCR primers were as follows: GLP-1R, sense 5′-ATCCACCTGAACCTGTTTGC-3′, anti-sense 5′-GCAGTATTGCATGAGCAGGA-3′; GAPDH, sense 5′-ACCACAGRCCATGCCATCAC-3′; anti-sense 5′-TCCACCACCCTGTTGCTGTA-3′. The amplification was performed in 35 cycles of 95°C for 30 s, 55°C for 30 s, and 72°C for 30 s. The PCR products were analyzed on 1.5% agarose gel electrophoresis.

### Western blot analysis

Cells were harvested by scraping in RIPA buffer (50 mM Tris-Cl, pH 7.4, 150 mM NaCl, 1 mM EDTA, 1% NP-40, 1 mM NaF, 1 mM Na_3_VO_4_, and 1 mM PMSF). Protein samples were separated by SDS-PAGE on 6%–15% acrylamide gels and transferred to PVDF membranes. The membranes were blocked with 5% skim milk in Tris-buffered saline containing 0.1% Triton X-100 for 1 h at room temperature. The membranes were incubated overnight at 4°C with specific primary antibodies and then hybridized with the secondary antibodies conjugated to horseradish peroxidase (HRP) for 1 h at room temperature. The reactions were detected by exposure to x-ray film after the application of the West-Q Chemiluminescent Substrate Plus Kit (GenDEPOT). The primary antibody dilutions were as follows: α-collagen I (1:2000, sc-28657, Santa Cruz), α-SMA (1:3000, A2547, Sigma-Aldrich), CTGF (1:1000, ab6992, Abcam), GLP-1R (1:250, ab39072, Abcam), and β-actin (1:5000, A5441, Sigma-Aldrich).

### [^3^H]-Thymidine uptake

PSCs were seeded in a 96-well plate at 3 × 10^3^ cells/well in DMEM containing 10% FBS and incubated at 37°C in a humidified 5% CO_2_ atmosphere. PSC proliferation was measured by [^3^H]-thymidine uptake. After serum starvation for 24 h, PSCs were cultured for 1, 2, and 3 days and pulsed with 1 μCi [^3^H]-thymidine (NET027, 1 mCi/mL, PerkinElmer) added to each well at 18 h before harvesting. The incorporation of [^3^H]-thymidine into the DNA was measured using a micro-β-scintillation counter (1450 LSC & Luminescence Counter, Perkin Elmer Korea).

### Measurement of Ang II and TGF-β1 production

To evaluate the effects of Ex-4 on Ang II production in high glucose-activated PSCs, the culture medium was collected for Ang II RIA assays (Buhlmann) after 6 h. The levels of TGF-β1 in the PSC supernatants were quantified at 1, 2, and 3 days using enzyme immunoassays according to the manufacturer’s protocols (R&D Systems). All culture media were supplemented with 10 nM angiotensinogen during the incubation period.

### Measurement of ROS

To measure the production of ROS, the treated cells were loaded with 5 μM dichloro-dihydrofluorescein dictate (DCF; Molecular Probes, Eugene, OR, USA) and incubated for 30 min at 37°C, 5% CO2. As a positive control, the cells were treated with 50 μM H_2_O_2_ for 30 min. The cells were washed and analyzed using a scanning fluorometer. The excitation and emission wavelengths were set to 490 and 535 nm, respectively.

### Statistical analysis

The results are expressed as the mean ± SE. All experiments were performed at least 3 times, and similar results were obtained in each replicate. The differences between groups were evaluated by ANOVA, followed by Fisher’s test for post-hoc analysis. P values < 0.05 were considered statistically significant.

## Results

### Ex-4 changes in non-fasting glucose level and body weight and glucose tolerance test in an animal model of T2DM

**Fig 1:**
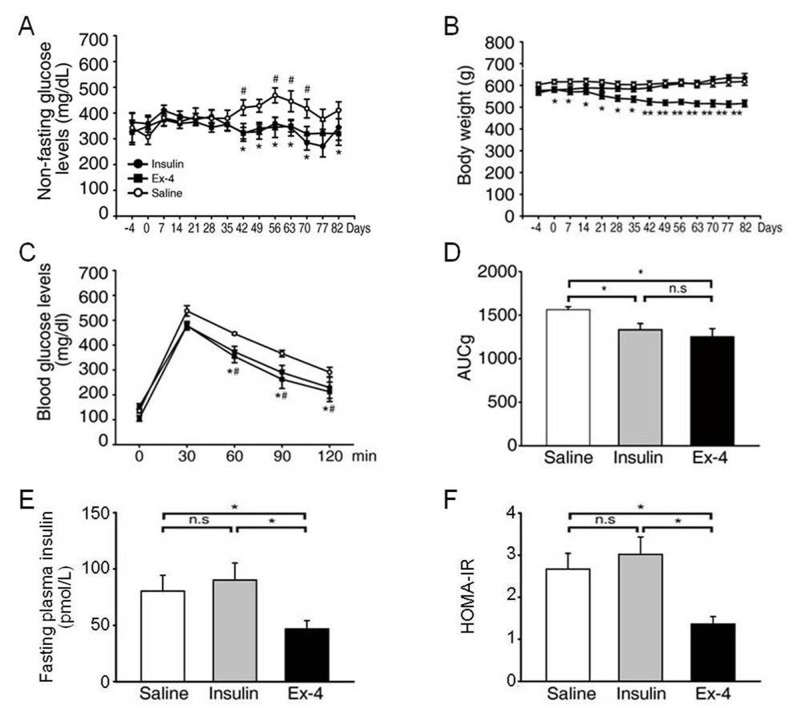
Effects of Ex-4 on OLETF rats. OLETF rats were randomly allocated into a saline- (n = 10) and insulin- (n = 10) injected control group or an Ex-4-injected group (n = 10) according to non-fasting glucose concentration. OLETF rats were injected with saline, Ex-4 or insulin for 12 weeks. Non-fasting plasma glucose (A) and body weight (B) in OLETF-treated for 12 weeks. At 12 weeks after injection, the diabetic rats were injected ip with 20% glucose. Blood samples were collected at 30, 60, 90, and 120 min after the injection. We then performed an IPGTT (C) and measured the area under the glucose curve (AUCg) (D), fasting insulin (E) and HOMA-IR (F). HOMA-IR was calculated as fasting insulin (mU/L) x fasting glucose (mmol/L)/ 22.5. The data represent mean ± SE (*, P<0.05 Ex-4 vs. Saline; #, P<0.05 Insulin vs. Saline).

We examined whether the administration of Ex-4 reduces islet fibrosis by examining the effects of Ex-4 in an animal model of chronic hyperglycemia. We maintained similar blood glucose levels in the Ex-4-treated and insulin-treated groups to evaluate the effect of Ex-4 on fibrosis repression, independent of its glucose-lowering effect. The random glucose levels in the insulin- and Ex-4-treated groups were significantly lower than those of the saline-treated group, without significant difference between the two treatment groups Fig 1A). After 12 weeks of treatment, body weight was significantly reduced only in the Ex-4-treated group (Fig 1B). The intraperitoneal glucose tolerance test results showed that the glucose values from 60 to 120 min after glucose loading were significantly lower in the Ex-4- and insulin-treated groups than in the saline-treated group (Fig 1C). Moreover, the mean areas under the glucose value curves were significantly higher in the saline-treated group than in the Ex-4- and insulin-treated groups (Fig 1D). In addition, fasting insulin level and HOMA-IR (Homeostatic model assessment- Insulin Resistance) index, a quantitative analysis to measure insulin resistance, were significantly lower in Ex-4-treated OLETF rat group compared to insulin- and saline-treated OLETF rat group (Fig 1E and 1F).

### Ex-4 attenuates the destructive changes in pancreatic islets in an animal model of T2DM

**Fig 2:**
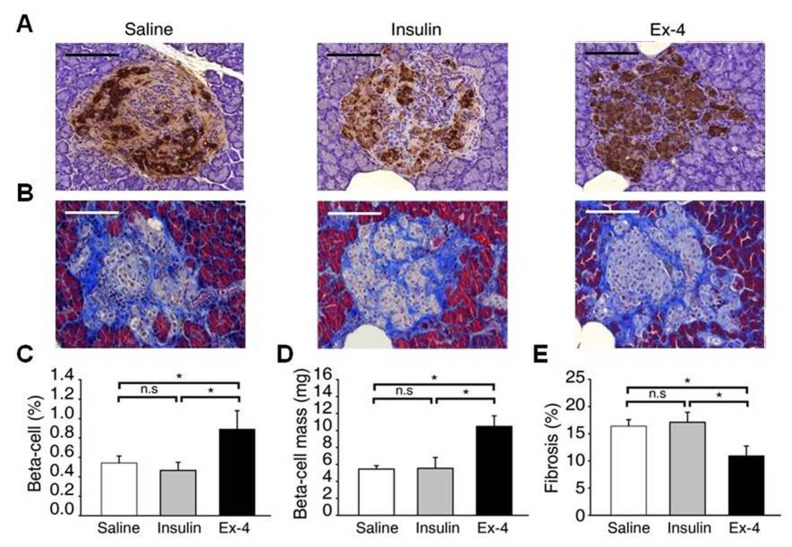
Effect of Ex-4 on pancreatic β-cell mass and islet fibrosis. (A) Representative images of OLETF rats-treated with saline, insulin or Ex-4 stained for insulin. (B) The degree of islet fibrosis was measured using trichrome staining. (C, D) β-cell (%) and β-cell mass were quantified in insulin-stained pancreas section. (E) The fibrotic area of islet after trichrome staining was quantitated by pixel counting and presented as a percentage (means **±** SE, *P < 0.05). Images shown are in the same magnification (x200).

Insulin showed a lower intensity in the saline- and insulin-treated groups, whereas it showed a consistent pattern in a large proportion of the islets of the Ex-4-treated group (Fig 2A). The relative volume of β-cells was significantly increased in the Ex-4-treated group (Fig 2C), and the β-cell mass, which was calculated from the relative volume of β-cells and the weight of the pancreas, was also increased in the Ex-4-treated group (Fig 2D). As demonstrated by trichrome staining, islet fibrosis within the islets was significantly increased in the saline- and insulin-treated OLETF rats. However, it was dramatically reduced in the Ex-4-treated group (Fig 2B), as revealed by the percent area stained per islet section (Fig 2E).

### Ex-4 suppresses high glucose-induced α-SMA expression and ROS production

**Fig 3:**
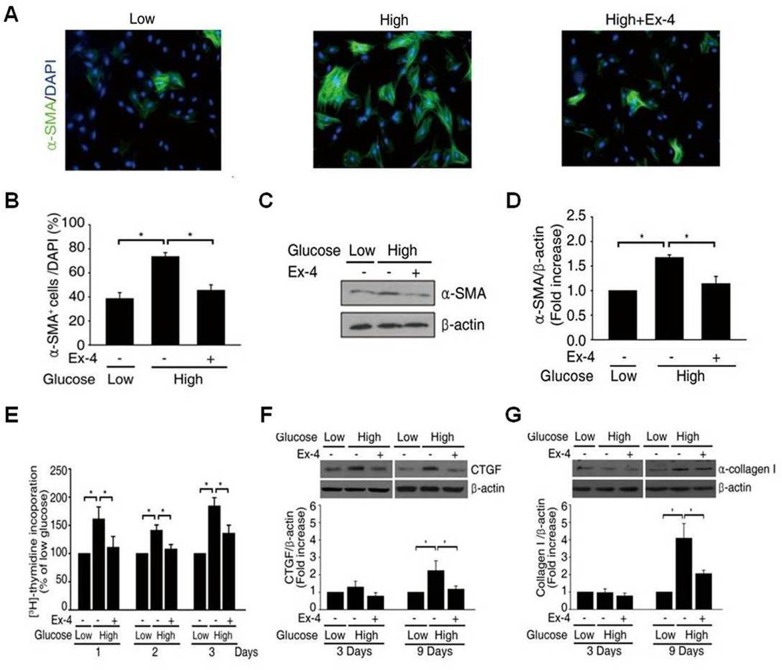
Effect of Ex-4 on α-SMA expression and proliferation. (A, C) immunofluorescence and western blots for α-SMA in rat PSCs treated with low, high glucose and/or Ex-4 for 3 days. (B, D) Quantitations of immunofluorescence and western blots normalized to β-actin or DAPI. (E) [^3^H]-thymidine incorporation significantly decreased after 1, 2 and 3 days in high glucose and Ex-4 compared with high glucose (n = 3 Separate cell preparations; *, P<0.05). (F, G) CTGF and α-collagen I protein were determined by western blotting, and quantitative analysis of CTGF and α-collagen I expression using densitometry (n = 3 separate cell preparations; *, P<0.05; **, P<0.005).

Previous studies have shown that high glucose stimulates α-SMA expression [22]. As shown in Fig 3A, a high glucose concentration significantly increased α-SMA expression after 72 h, whereas Ex-4 suppressed this effect. These data were quantified and expressed as a percentage of α-SMA^+^ cells in the total cells population (Fig 3B). We also examined whether Ex-4 treatment affects the high glucose-induced activation of PSCs by quantifying the expression of α-SMA using immunoblotting (Fig 3C and 3D). The formation of stress fiber-like structures was increased by high glucose, and the expression of α-SMA was reduced in Ex-4-treated PSCs. To investigate the effect of Ex-4 on high glucose-induced cell proliferation, we measured [^3^H]-thymidine incorporation in PSCs. Ex-4 treatment significantly reduced this glucose-induced proliferation after 24 h of incubation, and this effect was sustained over 72 h (Fig 3E). The expression of ECM proteins such as CTGF and α-collagen I, in response to high glucose was confirmed by western blotting the expression of CTGF and α-collagen I after 9 days in high glucose were significantly reduced by Ex-4 (Fig 3F and 3G).

### Ex-4 attenuates high glucose-induced ECM protein synthesis via the Ang II–TGF-β pathway

**Fig 4:**
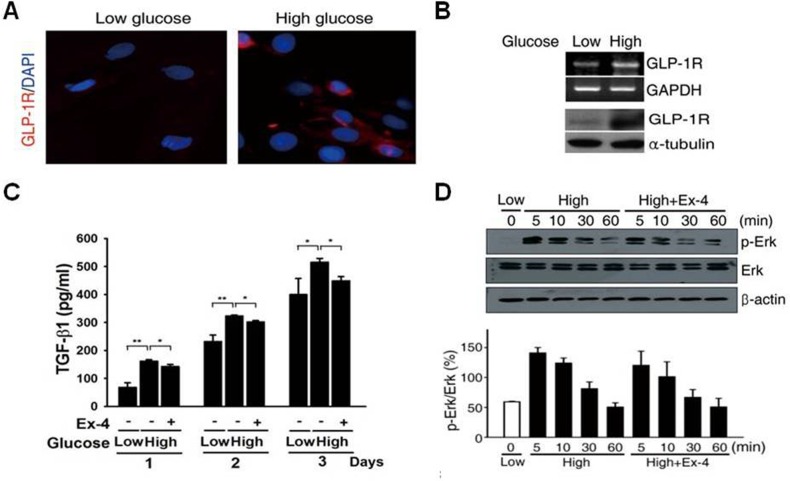
Effect of Ex-4 on high glucose-induced ECM protein synthesis. (A, B) GLP-1R expression confirmed using by immunofluorescence, RT-PCR, and western blot for PSCs 5 days after seeding. (C) TGF-β1concentration measurement in medium at 1–3 days. (D) Representative western blot for p-ERK in rat PSCs high glucose concentration from 5 min to 60 min and quantified using densitometry (n = 3 separate cell preparations; *, P<0.05; **, P<0.005).

**Fig 5:**
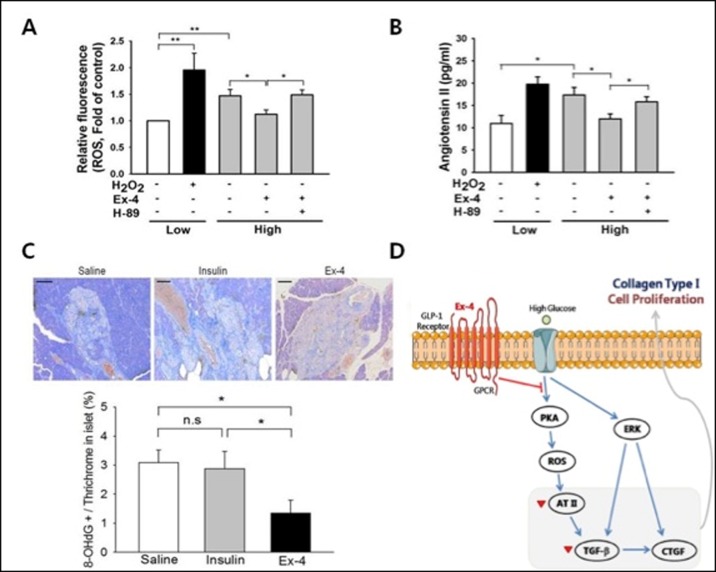
Effect of Ex-4 on high glucose-induced ROS production. (A) DCF fluorescence for measurement of ROS generaion was quantified using a scanning fluorometer. Magnification 100×. (B) Ang II concentration was measured by radioimmuoassay in medium at 6 h. (C) 8-OHdG positive cells (brown) were examined in the fibrotic area stained with trichrome (blue) of islets per groups. Quantified data shows that 8-OHdG positive cells were significantly decreased in Ex-4 group compared to other groups. The values represent the mean ± S.E of 5 experiments and are expressed as percent of LG value. (*, p<0.05 versus low glucose. **, p<0.001 versus low glucose.) (D) See the last paragraph of Results and Discussion for details.

Immunocytochemistry showed that GLP-1R localized to the membrane and cytoplasm in PSCs (Fig 4A). GLP-1R expression was detected under both low and high glucose conditions. Furthermore, GLP-1R mRNA and protein expressions (Fig 4B) were significantly increased under high glucose conditions compared with low-glucose conditions. These results demonstrate that GLP-1R is expressed in PSCs and that its expression is affected by glucose concentrations. To measure ROS production, PSCs were exposed to DCF, a fluorescent marker of cellular oxidant production [16]. We measured the TGF-β1 concentration in same conditions to determine whether Ex-4 reduces TGF-β1 production in high glucose-activated PSCs high glucose markedly stimulated the production of TGF-β1 in a time-dependent manner, and this stimulation was decreased by Ex-4 treatment (Fig 4C). We also examined the effect of Ex-4 on ERK activation by western blotting and found that high glucose level increased ERK activation; however, no effects of Ex-4 treatment on ERK phosphorylation were observed (Fig 4D). Fig 5 shows that, similar to H_2_O_2_, high glucose, induced a higher fluorescent intensity compared to the low glucose, which indicated the induction of ROS production by high glucose. Interestingly, Ex-4 treatment significantly reduced ROS production. H-89, a PKA inhibitor completely blocked the inhibitory effect of Ex-4 on high glucose-induced ROS production (Fig 5A). We also measured Ang II secretion into the medium and found that the Ang II concentration was dramatically increased in the presence of high glucose and H_2_O_2_; however, treatment with Ex-4 under high glucose led to a marked attenuation of Ang II levels. This effect of Ex-4 inhibited by H-89 treatment (Fig 5B). Islet expression of 8-OHdG in the saline- and insulin-treated groups was significantly higher than that of Ex-4-treated OLETF rats. Ex-4 treatment for 12 weeks effectively abolished the percentage of 8-OHdG-positive nuclei/thrichrome in islets (Fig 5C).

In the present study, we examined the effect of Ex-4 on ECM protein synthesis in glucose-activated PSCs. Although a high level of glucose enhanced the ERK-phosphorylation, which was unchanged by Ex-4 treatment (Fig 5D).

## Discussion

The current study demonstrates that treatment with Ex-4 dramatically attenuates oxidative stress, decreases pancreatic islet fibrosis within the islets and preserves islet mass. We compared the anti-fibrotic effects of two anti-diabetic medications with different glucose-lowering mechanisms, insulin and Ex-4, in this experiment. Insulin generally attenuates hyperglycemia by lowering postprandial hyperglycemia. Glycemic status was not different between insulin- and Ex-4-treated rats throughout the observation period. Despite similar glucose-lowering actions, Ex-4 showed a greater decrease in fibrosis and a greater increase in islet β-cell mass than insulin treatment. In addition, fasting insulin and HOMA-IR were decreased by Ex-4 treatment. Therefore, we suggest that Ex-4 treatment ameliorates insulin resistance and has additional benefits on the progression or prevention of diabetic fibrosis in type 2 diabetes. These findings are important because pancreatic β-cell dysfunction, characterized by decreased insulin secretion due to functional defects of the β-cells and/or insufficient β-cell mass, plays an important role in the pathogenesis of type 2 diabetes [23, 24].

Previously, we found conspicuous islet fibrosis with destroyed islet construction, which was accompanied by α-SMA-positive cells, a specific marker of PSCs, in an advanced type 2 diabetes mellitus animal model without evidence of pancreatitis. OLETF rats with diabetic progression display severe islet destruction because of fibrosis, which is accompanied by increased expression of α-SMA in the pancreas, especially surrounding the destroyed islets [25]. In addition, the fibrotic change and extracellular matrix (ECM) production of pancreatic islets in OLETF rats was significantly attenuated by Ramipril treatment [8]. In addition, we reported that high glucose increased ECM production in cultured pancreatic stellate cells via the renin-angiotensin system [25]. GLP-1 and Ex-4 are known to activate multiple signal transduction pathways, such as cAMP/PKA, PI-3 kinase and MAPK, which lead to increased islet mass and beta cell growth [17]. We hypothesized that a GLP-1R agonist would modulate various signaling pathways such as cAMP/PKA and the production of ROS and ECM related to islet fibrosis. In this study we used an OLETF animal study and an in vitro study of PSCs from SD rats to investigate our hypothesis. In the OLETF rat study, we observed that long-term treatment with Ex-4 improved glucose tolerance and reduced fibrosis in the islets. To clarify the role of the Ex-4 mediated signaling pathway (such as cAMP/PKA) we used an in vitro culture model of PSCs activated by high glucose. Ideally, we should have used PSCs from OLETF rats at two specific time points, before and after the Ex-4 treatment. However, because it is difficult to obtain large numbers of PSCs from pancreatic tissues, we isolated PSCs from the pancreases of SD rats.

Activation of the PKA pathway by Ex-4 plays a role in oxidative stress (or ROS production) and Ang II production. PSCs showed a markedly increased ECM protein synthesis rate in response to high glucose conditions. Hyperglycemia stimulates the activation of PSCs via the activation of Ang II and TGF-β1 signals [26] and the induction of ERK 1/2 phosphorylation [22]. GLP-1R activation results in the amelioration of ROS production through Epac [27]. In this study we did not investigate the possible involvement of Epac and GLP-1R in the in vitro model of PSCs and this is something that needs to be investigated to further clarify the mechanism [27].

In this study, we showed that high glucose-activated pancreatic stellate cells may invoke two independent signaling pathways (Fig 5D): ^1)^ the ERK1/2 [28] and p38 MAPK (mitogen-activated protein kinase) pathway [25] and ^2)^the Ang II (angiotensin type II receptor) signaling pathway through the production of ROS induced by chronic high glucose exposure [22]. These pathways ultimately lead to TGF-β1 production and the expression of CTGF, an important downstream mediator of TGF-β1 activity [29]. Wenbin and his colleagues observed a direct effect of Ex-4 on TGF-β1 and CTGF expression by using a specific adenylate cyclase inhibitor and reported that Ex-4 has an anti-fibrotic effect [30]. In our study, we observed that Ex-4 inhibited TGF-β1 by a signaling cascade initiated by reducing high-glucose-mediated ROS production and AT-II. This suggests that Ex-4 directly inhibits the expression of TGF-β1 by reducing high-glucose-mediated ROS production and AT-II.

In conclusion, our data demonstrate that Ex-4 significantly reduced Ang II and TGF-β1 production by inhibition of ROS production but not ERK phosphorylation. This inhibitory effect of Ex-4 was largely related with cAMP/PKA signaling pathway. Thus these results suggest that Ex-4 may be useful not only as an anti-diabetic agent but also as an anti-fibrotic agent in type 2 diabetes.

## Supporting Information

S1 File PSC RAW data. (XLSX)

S2 File psc body weight & blood glucose (XLS)

## Abbreviations

PSCs: Pancreatic stellate cells
OLETF rats: Otsuka Long-Evans Tokushima Fatty (OLETF) rats
Ex-4: Exendin-4
TGF β1: Transforming growth factor beta
ATII: Angiotensin II
α-SMA: α-smooth muscle actin
